# Self-compassion and music performance anxiety: the mediating role of fear of negative evaluation and the moderating role of psychological resilience

**DOI:** 10.3389/fpsyg.2026.1791913

**Published:** 2026-05-20

**Authors:** Lei Wang, Xiongying Li

**Affiliations:** 1 College of Arts, Lanzhou University, Lanzhou, Gansu, China; 2 Higher Education Research Institute, Lanzhou University, Lanzhou, Gansu, China

**Keywords:** fear of negative evaluation, music major undergraduates, music performance anxiety, psychological resilience, self-compassion

## Abstract

**Background:**

Music performance anxiety is common in high-pressure performance contexts and may relate to both performance quality and psychological well-being, yet empirical research in this field remains limited. To address the need for effective strategies that reduce music performance anxiety among college students and to extend current knowledge, this study draws on emotion regulation theory and the risk-protection framework to examine the association between self-compassion and music performance anxiety, the mediating role of fear of negative evaluation, and the moderating role of psychological resilience.

**Method:**

We analyzed data from 971 college students using SPSS 26.0 to test the proposed hypotheses.

**Results:**

The results showed a significant negative association between self-compassion and music performance anxiety, with fear of negative evaluation serving as a partial mediator. The mediating effect accounted for 41.667% of the total effect. In addition, psychological resilience negatively moderated the associations of self-compassion and fear of negative evaluation with music performance anxiety but did not moderate the link between self-compassion and fear of negative evaluation.

**Conclusion:**

This study contributes to understanding the link between self-compassion and performance anxiety, focuses on the mediating role of fear of negative evaluation, and emphasizes the protective role of psychological resilience. It also offers both theoretical and practical insights for creating emotional regulation and intervention strategies for students in the arts.

## 1 Introduction

Music performance anxiety (MPA), a widespread concern among musicians, is often linked to various aspects of their mental health, performance quality, and overall quality of life (Kenny, 2011). MPA typically manifests as excessive tension during performance, fear of making mistakes, and heightened worry about others’ evaluations, and severe symptoms may interfere with normal performance functioning (Kenny, 2005). These challenges are often more pronounced among college students majoring in music performance, who may have relatively limited performance experience (Barros et al., 2022). MPA has received growing attention in the academic literature, with studies examining its manifestations and correlates across different groups, such as amateur musicians, adolescents, and university students (Ananias de Lima et al., 2024; Spahn et al., 2023). Existing research has primarily examined psychological factors that relate to MPA. Findings indicate that self-esteem, psychological capital, and flow experience are associated with MPA (Jiang and Tong, 2024), whereas self-perception and emotion regulation abilities have also been linked to MPA (Yan et al., 2025). External factors such as social support, performance setting, and audience may also influence MPA by shaping individuals’ internal psychological resources (Kenny, 2026; Zhang and Jenatabadi, 2024).

Recent studies suggest that emotion regulation plays a central role in understanding MPA (Kaleńska-Rodzaj, 2021). Self-compassion (SC), recognized as an effective emotion regulation strategy, is associated with lower emotional distress and reduced psychological burden (Neff and Germer, 2013). Fear of negative evaluation (FNE) has been identified as an important component of music performance anxiety across different performance contexts (Nicholson et al., 2015). In addition, individuals with high psychological resilience (PR) tend to experience less anxiety-related distress, as they are more inclined to approach stressful events with adaptive and positive coping strategies (Chuning et al., 2024). Given that PR is associated with lower anxiety-related distress, it is worthwhile to investigate whether the connection between SC and MPA varies across different levels of PR. However, research examining the link between SC and MPA remains limited, and few studies have explored the mechanisms underlying this association. Drawing on emotion regulation theory (Gross, 2002) and the protective–vulnerability framework (Fergus and Zimmerman, 2005), this study investigates how SC relates to MPA through FNE and examines whether PR moderates these associations. The findings aim to deepen the current understanding of MPA and provide theoretical and practical insights for promoting psychological wellbeing among music performers.

## 2 Literature review

### 2.1 Theoretical foundation

This study is based on emotion regulation theory (Gross, 2002) and the risk–protection framework (Fergus and Zimmerman, 2005). Emotion regulation theory proposes that individuals actively use cognitive and behavioral methods to monitor, interpret, and manage the emergence, processing, and expression of emotions (Gross, 2002). SC emphasizes treating oneself with kindness, understanding, and acceptance when facing setbacks (Neff, 2003). Prior research indicates that SC is associated with less negative emotional reactivity to stressful or unpleasant events (Bluth et al., 2016; Leary et al., 2007) and with lower levels of self-criticism (Joeng and Turner, 2015). In music performance settings, students often show heightened sensitivity to mistakes and external evaluation, and FNE is closely associated with performance anxiety (Kenny et al., 2013). From an emotion regulation perspective, SC may lessen excessive self-focus on perceived shortcomings, reduce the perceived threat of external judgment, and thereby be associated with lower FNE. Thus, FNE may function as an explanatory pathway in the association between SC and MPA.

According to the risk–protection framework (Fergus and Zimmerman, 2005), psychological outcomes are influenced by the dynamic relationship between risk factors and protective resources. Risk factors increase the likelihood of negative emotions or psychological distress, whereas protective resources buffer these risks and support more stable emotional functioning (Fergus and Zimmerman, 2005). In the present study, FNE represents a typical risk factor. It can heighten students’ tension, worry, and expectations of failure in performance situations, thereby being associated with higher levels of performance-related anxiety (Cooper and Brownell, 2020). In contrast, PR represents a crucial protective resource that reflects individuals’ capacity to maintain emotional balance, recover from stress, and adapt effectively when encountering challenges or evaluative pressures (Ellis et al., 2017). Evidence suggests that individuals with elevated PR tend to maintain emotional stability, preserve self-efficacy, and exhibit fewer heightened reactions to social evaluative stress (Berdida et al., 2023; Han and Wang, 2022). Based on the risk–protection framework (Fergus and Zimmerman, 2005), PR may buffer the association between FNE and MPA.

Integrating these two theoretical perspectives provides the foundation for the present study. SC may foster adaptive emotion regulation, reduce excessive concern about negative evaluation, and be related to lower levels of MPA. At the same time, PR, as a protective factor, may weaken the association between FNE and performance anxiety. This integrated perspective offers a theory-based explanation for examining how SC, FNE, and PR may be related to performance anxiety and underscores the buffering role of protective resources in high-risk contexts, thereby providing a clearer basis for understanding music students’ emotional experiences and coping processes.

### 2.2 Self-compassion and music performance anxiety

SC refers to adopting a nurturing and accepting attitude when encountering setbacks, failures, or negative emotional experiences, rather than falling into self-criticism, denial, or shame (Neff, 2003). Unlike traditional self-esteem, SC does not depend on external achievements or social comparison. Instead, it reflects a stable, self-care–oriented emotional response pattern that emphasizes cognitive acceptance of personal suffering and emotional support toward oneself (Neff, 2023). Within the framework of emotion regulation theory (Fergus and Zimmerman, 2005; Gross, 2002), SC may be understood as an adaptive emotion regulation strategy. It promotes positive cognitive reappraisal, reduces over-identification with negative emotions, and enhances emotional awareness, enabling individuals to respond more evenly to stress or failure (Diedrich et al., 2014). Studies have shown that SC is linked to a reduction in both excessive self-criticism and threat-based interpretations, thereby reducing the amplification of negative emotions and relating to lower symptoms of social anxiety (Harwood and Kocovski, 2017), competitive anxiety (Casali et al., 2022; Jansen et al., 2021), and trait anxiety (Hodes et al., 2022). Studies have demonstrated that students with elevated SC levels generally report experiencing lower MPA (Farley and Kelley, 2023; Paese and Egermann, 2024; Regier et al., 2025). For example, Farley and Kelley (2023) found that music performance students with elevated SC, particularly those excelling in self-kindness, reported experiencing lower MPA. Using semi-structured interviews with music professionals, Paese and Egermann (2024) found that performers who practiced loving-kindness and SC meditation tended to adopt compassionate attitudes and non-judgmental self-narratives. These practices were associated with reduced negative emotions stemming from fear of rejection, which, in turn, corresponded to lower MPA. Although existing studies consistently document a significant association between SC and MPA, research on the mechanisms underlying this relationship remains limited. Current work has not fully explored the pathways through which SC may relate to performance-related anxiety, indicating the need for further investigation.

### 2.3 The mediating role of fear of negative evaluation

FNE refers to the anxiety individuals experience regarding others’ assessments of them, particularly the distress triggered by the possibility of being judged negatively, their tendency to avoid situations involving evaluation, and the anticipation of unfavorable judgments (Watson and Friend, 1970). As a typical threat bias in social cognitive processing, FNE reflects an exaggerated interpretation of the relationship between one’s performance and others’ judgments (Weeks et al., 2008). From the perspective of emotion regulation theory, SC is considered a positive psychological resource that promotes cognitive reappraisal, reduces over-identification with emotions, and enhances emotional balance (Neff, 2003). Research consistently demonstrates that SC can buffer psychological stress in self-evaluative situations and is correlated with lower FNE (Liu et al., 2022; Long and Neff, 2018; Mosewich et al., 2011; Werner et al., 2012; Zhang et al., 2019). In a study by Werner et al. (2012), individuals diagnosed with social anxiety disorder showed significantly lower SC and reported higher FNE compared to the healthy control group. Liu et al. (2022) also reported that SC relates to reduced FNE and, in turn, lower social anxiety, which corresponded to decreased loneliness among adolescents. Some studies further suggest that FNE may reduce SC. For example, Huang and Wang (2024) found among Chinese college students that both fear of negative and positive evaluation were associated with lower levels of SC. However, these associations have not yet been explored in music performance students.

According to the risk–protection framework, FNE may function as an important risk factor for anxiety responses, as it tends to intensify negative emotions (Fergus and Zimmerman, 2005). Recent studies consistently show that FNE is an important factor associated with social anxiety and performance anxiety, and it has been linked to emotional responses, attentional allocation, and behavioral regulation (Carleton et al., 2011; Gomez et al., 2023). In the domain of music performance, FNE has also been identified as a key factor associated with elevated MPA (Gök and Yalçinkaya-Alkar, 2024; Kenny et al., 2013; Nicholson et al., 2015). For example, Kenny et al. (2013) found among orchestral musicians that both state anxiety and FNE predicted MPA. Gök and Yalçinkaya-Alkar (2024) further reported that perfectionistic tendencies were linked to higher FNE in musicians, which corresponded to higher levels of MPA. A recent review by Nicholl and Abbott (2024) also highlighted FNE as a central mechanism connecting social anxiety and MPA. Taken together, FNE is not only an important risk factor for performance anxiety but may also serve as a key mediating variable between positive psychological resources and anxiety-related responses. Although existing studies have established a negative relationship between SC and FNE and emphasized FNE’s role in MPA, no empirical research has yet examined if FNE mediates the connection between SC and MPA. Therefore, further research is needed to clarify the psychological processes associated with MPA.

### 2.4 The moderating role of psychological resilience

PR refers to individuals’ ability to cope with stress and adversity, providing internal support and regulation when they encounter challenges (Masten, 2001). According to the risk–protection framework, PR functions as a key positive psychological resource. This process facilitates emotional recovery, ensures cognitive flexibility and emotional balance even under high-pressure circumstances, and may lessen the harmful effects of risk factors on mental health (Fergus and Zimmerman, 2005). Therefore, PR may serve as a moderator in this study.

First, PR may moderate the association between SC and FNE. SC is generally linked to reduced self-criticism and threat-based cognitions, which correspond to lower levels of FNE (Long and Neff, 2018). However, individuals with high resilience tend to possess stronger emotional recovery and cognitive flexibility when facing potential negative evaluation. Even with relatively low SC, they may still maintain stable self-evaluations and avoid excessive focus on external judgments. Studies have shown that people with high resilience are more prone to apply positive reappraisal in social contexts where evaluation occurs, which in turn reduces the intensity of FNE (Sünbül and Yerin Güneri, 2019; Hu et al., 2015). Thus, PR may moderate the association between SC and FNE, such that the association may be stronger at higher levels of PR and weaker when PR is limited.

Second, PR may moderate the association between SC and MPA. SC is associated with lower self-criticism, reduced shame, and more adaptive emotion regulation, all of which relate to lower levels of anxiety (Neff and Germer, 2018). Studies indicate that PR helps individuals maintain stable emotional states and self-efficacy under performance pressure, thereby relating to lower anxiety responses (Du et al., 2025; Kegelaers et al., 2021). Higher levels of PR may be associated with a stronger negative association between SC and anxiety, whereas lower levels of PR may be associated with a weaker association between these variables. Under lower PR conditions, anxiety may remain relatively elevated even across different levels of SC.

Third, PR is a particularly salient protective factor that may moderate the association between FNE and MPA. As a cognitive risk factor, FNE is linked to heightened perceptions of threat in performance contexts and to anxious responses (Carleton et al., 2007). PR can reduce threat-based interpretations, enhance psychological endurance, and promote more effective stress-coping strategies, thereby weakening the association between FNE and anxiety (Arbinaga, 2023). Research indicates that individuals who exhibit high resilience tend to experience lower levels of anxiety in evaluative situations, such as social or performance contexts, even in the presence of FNE (Killgore et al., 2020; Sun et al., 2025). For example, Pompili et al. (2024) found that adolescents with high FNE showed stronger problematic drinking and anorexic behaviors only when their resilience levels were low; those with high resilience did not exhibit this pattern. In this way, PR may serve as a buffer, diminishing the influence of risk factors on the emotional experience of anxiety. In summary, PR may moderate all three pathways involving SC, FNE, and MPA. By enhancing emotion regulation, facilitating positive cognitive processing, and strengthening coping strategies, PR may influence how individuals appraise evaluative threats and, consequently, the extent to which they experience performance-related anxiety.

### 2.5 The current study

**FIGURE 1:**
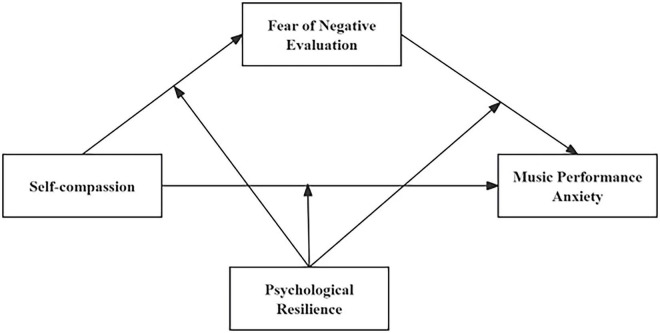
Hypotheses and research model.

Building on the above literature review, this study draws on emotion regulation theory (Gross, 2002) and the risk–protection framework (Fergus and Zimmerman, 2005) to examine the association between SC and MPA, as well as the roles of FNE and PR in this relationship. Therefore, we propose the following hypotheses and the research model illustrated in Figure 1.

*H1*: SC is negatively associated with MPA.

*H2*: FNE mediates the association between SC and MPA.

*H3a*: PR moderates the association between SC and MPA, such that the negative association between SC and MPA is stronger at higher levels of PR.

*H3b*: PR moderates the association between SC and FNE, such that the negative association between SC and FNE is stronger at higher levels of PR.

*H3c*: PR moderates the association between FNE and MPA, such that the positive association between FNE and MPA is weaker at higher levels of PR.

## 3 Materials and methods

### 3.1 Procedures

The Ethics Committee of Lanzhou University approved this study, which was conducted at several universities in China. All steps followed the ethical principles of the Declaration of Helsinki, from participant recruitment to data collection and analysis. We used a cross-sectional design and administered the survey through the online platform Wenjuanxing in October 2025. Participants were recruited using convenience sampling from colleges and majors related to music performance at several universities in China.

During the recruitment process, the research team first contacted administrators from relevant colleges to obtain information on class arrangements and course schedules for music performance majors. Based on accessibility and institutional support, 30 classes were approached for participation. During non-class hours, research staff visited these classes and invited available students who agreed to participate to complete the survey. Before distributing the questionnaire, trained research assistants read aloud the informed consent form, which outlined the study purpose, estimated completion time (about 10 minutes), data usage, confidentiality procedures, and participants’ ability to withdraw without any academic repercussions. Only non-sensitive demographic information relevant to the study, such as grade level and major, was collected. After providing informed consent, participants were guided to access the Wenjuanxing platform^1^ to complete the questionnaire.

### 3.2 Participants

A total of 1,100 participants were invited, and 1,003 questionnaires were collected. After data cleaning, we removed 17 incomplete questionnaires and 15 questionnaires with inattentive responding (e.g., selecting the same option for most items despite the presence of reverse-coded items, or extremely long or short completion times). 971 valid responses were obtained for the final dataset, resulting in an effective response rate of 88.273%.

Demographic information for the participants is presented in Table 1. Among the 971 participants, the proportion of women was higher (705 students, 72.61%), which is consistent with the gender distribution typically observed in music performance programs in China (Cui et al., 2024; Yang et al., 2025). The majority of participants were aged 18–22, making up 68.07% of the sample. The distribution across grade levels was relatively balanced, with first- and second-year students accounting for the largest proportions (30.28 and 35.02%, respectively). Regarding major, students specializing in piano and vocal performance made up the largest groups, followed by those majoring in traditional Chinese music and orchestral instruments. In terms of years of musical study, most participants reported between 1 and 6 years of training (64.98%), and the majority had studied music for 3 years or more.

**TABLE 1:** Frequency analysis of demographic variables.

Type	Classification	Frequency	Percentage
Gender	Male	266	27.39%
	Female	705	72.61%
Age	18∼20 years old	351	36.14%
	20∼22 years old	310	31.93%
	Over 22 years old	310	31.93%
Grades	First year	294	30.28%
	Second year	340	35.02%
	Third year	170	17.50%
	Fourth year	167	17.20%
Major	Piano	283	29.15%
	Vocal music	247	25.44%
	Traditional Chinese music	88	9.06%
	Orchestral music	97	9.99%
	Music education	89	9.17%
	Contemporary popular music	78	8.03%
	Composition	89	9.17%
Duration of music study	1–3 years	289	29.76%
	4–6 years	342	35.22%
	7–9 years	167	17.20%
	Over 10 years	173	17.82%

*N* = 971.

### 3.3 Measures

The survey used in this study consisted of two parts. The first part collected demographic information, including age, grade level, and gender. The second part collected participants’ responses to the study variables. All instruments were selected from established and validated scales. Since the target population was Chinese college students, all items were presented in Chinese. For SC, FNE, and PR, previously validated Chinese versions were used. For MPA, as no well-established Chinese version of the K-MPAI suitable for the present sample was available, to ensure linguistic and conceptual equivalence, the scale was translated through the translation-back-translation procedure (Brislin, 1980).

SC was measured using the Self-Compassion Scale (SCS) developed by Neff (2003). This scale has been validated in prior studies and has demonstrated good reliability and validity among Chinese college students (Sheng et al., 2023; Zhao et al., 2023). The SCS contains 26 items rated on a five-point Likert scale and includes six dimensions: self-kindness (5 items), self-judgment (5 items), common humanity (4 items), isolation (4 items), mindfulness (4 items), and over-identification (4 items). Items in the Self-Judgment, Isolation, and Over-Identification dimensions were reverse-coded. In the present study, the Cronbach’s α for the SCS was 0.889.

MPA was measured using the Kenny Music Performance Anxiety Inventory (K-MPAI) developed by Kenny et al. (2004). This scale has been validated in prior studies and has shown strong psychometric performance among Chinese (Jiang and Tong, 2024). The K-MPAI includes 26 items rated on a seven-point Likert scale and contains three dimensions: biological vulnerability predispositions and early contextual/parental vulnerabilities (5 items), generalized psychological vulnerability (13 items), and specific triggering factors causing subsequent concerns about performance (8 items). Items 2, 9, 14, 19, 24, and 26 were reverse-coded. In this study, the Cronbach’s α for the K-MPAI was 0.921.

FNE was measured using the Brief Fear of Negative Evaluation Scale (BFNES) developed by Leary (1983). This scale has been validated in prior studies and has shown strong psychometric performance among Chinese college students (Lin et al., 2025). The instrument includes 12 items rated on a five-point Likert scale and adopts a single-factor structure. Items 2, 4, 7, and 10 were reverse-coded. In this study, the Cronbach’s α for the BFNES was 0.821.

PR was measured using the Connor–Davidson Resilience Scale (CD-RISC) developed by Campbell-Sills and Stein (2007). This scale has been validated in prior studies and has shown strong psychometric performance among Chinese college students (She et al., 2020). The scale contains 10 items rated on a five-point Likert scale and adopts a single-dimensional structure. In the present study, the Cronbach’s α for the CD-RISC was 0.786.

### 3.4 Statistical analysis

Using SPSS 26.0 and AMOS 26.0, data were analyzed in six steps. First, descriptive statistics were calculated for the continuous variables, and Pearson correlation analysis was performed to investigate the relationships among the study variables. Second, common method bias (CMB) was assessed through Harman’s one-factor test. To further evaluate the structural validity of the measurement model and assess the potential influence of method bias, confirmatory factor analysis (CFA) and an unmeasured latent method construct (ULMC) comparison were conducted using AMOS 26.0. Third, multicollinearity was checked using the variance inflation factor (VIF), with values under 3.3 suggesting that multicollinearity was not a significant issue (Shrestha, 2020). Gender, age, and grade level were included as covariates in the mediation and moderation analyses, and all variables were standardized before analysis. Forth, mediation analysis was conducted using the Bootstrap method in PROCESS Macro version 4.2 (Model 4), with 5,000 bootstrap resamples and 95% confidence intervals (CIs). The indirect effect was significant when the 95% CI excluded zero (Mackinnon et al., 2004; Mallinckrodt et al., 2006). Finally, the moderating role of PR was examined using PROCESS Model 59. For statistically significant moderation effects, Johnson–Neyman analysis was further conducted in PROCESS to identify the regions of significance, and the corresponding plots were created in Excel.

## 4 Results

### 4.1 Descriptive statistics and correlation analysis

The descriptive statistics for the main variables, including kurtosis (K), skewness (S), mean (M), standard deviation (SD), and Pearson correlation coefficients, are shown in Tables 2, 3. Participants reported relatively high levels of SC (M = 3.512) and PR (M = 3.560), while the levels of MPA (M = 3.773) and FNE (M = 2.443) were lower. The correlation results indicate that SC is negatively correlated with MPA (*r* = –0.668, *p* < 0.001) and FNE (*r* = –0.666, *p* < 0.001), while FNE is positively correlated with MPA (*r* = 0.679, *p* < 0.001). These correlation directions are consistent with the theoretical expectations.

**TABLE 2:** Descriptive statistics.

Variables	M ± SD	*K*	*S*
SC	3.512 ± 0.535	–1.048	–0.102
MPA	3.773 ± 0.723	–0.959	–0.031
FNE	2.443 ± 0.574	–0.733	0.197
PR	3.560 ± 0.582	–0.885	–0.111

*N* = 971. SC, Self-compassion; MPA, Music Performance Anxiety; FNE– Fear of Negative Evaluation; PR, Psychological Resilience; M, Mean; SD, Standard Deviation; K, Kurtosis; S, Skewness.

**TABLE 3:** Correlations among variables.

Variables	SC	MPA	FNE	PR
SC	1	1	1	1
MPA	–0.668 ***			
FNE	–0.666***	0.679***		
PR	–0.606***	–0.559***	–0.712***	

SC, Self-compassion; MPA, Music Performance Anxiety; FNE, Fear of Negative Evaluation; PR, Psychological Resilience.

***p < 0.001.

### 4.2 Common method bias

First, CMB was assessed using Harman’s one-factor test. The exploratory factor analysis extracted 13 factors with eigenvalues greater than 1, and the first unrotated factor accounted for 23.552% of the total variance, which was below the commonly used threshold of 40% (Podsakoff et al., 2003). These findings indicate that common method bias was not a significant issue.

Second, we performed CFA to further examine the structural validity of the measurement instruments. As shown in Table 4, the measurement model demonstrated good overall fit: χ^2^ = 3611.536, *df* = 2621, χ^2^*/df* = 1.378, RMSEA = 0.020, SRMR = 0.034, GFI = 0.912, AGFI = 0.907, CFI = 0.946, IFI = 0.946, and TLI = 0.944. All indices met commonly accepted criteria for structural equation modeling, indicating strong convergent validity and satisfactory model fit (Hu and Bentler, 1999). To further evaluate CMB, we constructed an Unmeasured Latent Method Construct (ULMC) model, which showed minimal differences in fit indices compared to the baseline model, confirming that CMB was not a significant issue (Fuller et al., 2015). This solid structural validity provides a reliable foundation for the subsequent analyses.

**TABLE 4:** CFA fit indices and comparison between baseline and ULMC models.

Model	χ *^2^*	*df*	χ *^2^/df*	RMSEA	SRMR	GFI	AGFI	CFI	IFI	TLI
Baseline value	3611.536	2,621	1.378	0.020	0.034	0.912	0.907	0.946	0.946	0.944
ULMC Model	2968.598	2,547	1.166	0.013	0.025	0.926	0.919	0.977	0.977	0.975
Absolute difference	–	–	–	0.007	0.009	0.014	0.012	0.031	0.031	0.031

ULMC, unmeasured latent method construct. Recommended criteria for acceptable model fit were χ*^2^/df* < 3, RMSEA < 0.06, SRMR < 0.08, and GFI, AGFI, CFI, IFI, and TLI > 0.90 (Hu and Bentler, 1999). For model comparison, differences smaller than 0.05 for RMSEA and SRMR and smaller than 0.10 for GFI, AGFI, CFI, IFI, and TLI were considered acceptable (Fuller et al., 2015).

### 4.3 Multicollinearity diagnostics

When MPA was the dependent variable, the VIF values were 1.909 for SC, 2.513 for FNE, and 2.211 for PR, all below the threshold of 3.3, indicating no serious multicollinearity (Shrestha, 2020).

### 4.4 Mediating effect analysis

The regression findings are presented in Table 5. The results showed that SC was significantly negatively associated with FNE (β = –0.668, *p* < 0.001) and MPA (β = –0.392, *p* < 0.001), indicating that students with higher levels of SC tended to report lower levels of FNE and MPA. FNE was significantly positively associated with MPA (β = 0.419, *p* < 0.001), suggesting that greater FNE to higher levels of MPA. The explanatory power of the models was relatively strong, with *R*^2^-values of 0.445 for FNE and 0.549 for MPA, indicating good model fit. In addition, none of the covariates reached statistical significance, so their coefficients are not reported in Table 4. Overall, these findings provide statistical support for further examining the mediating role of FNE in the association between SC and MPA.

**TABLE 5:** Regression results for the associations among the study variables.

Outcome variable	Predictor variable	β	SE	*T*	Bootstrap 95% CI		*R* ^2^	*F*
					LLCI	ULCI		
FNE	SC	–0.668	0.024	–27.723***	–0.715	–0.621	0.445	128.692
MPA	SC	–0.392	0.029	–13.466***	–0.449	–0.335	0.549	167.310
	FNE	–0.419	0.029	14.410***	0.362	0.476		

*N* = 971.

***p < 0.001. FNE, Fear of Negative Evaluation; SC, Self-compassion; MPA, Music Performance Anxiety.

As shown in Table 6, the total effect of SC on MPA was -0.672, and the direct effect was -0.392. When FNE was included as a mediator, the indirect effect was -0.280, accounting for 41.677% of the total effect, indicating a partial mediating effect. Therefore, both H1 and H2 were supported.

**TABLE 6:** The mediation effect.

Effect	Path	β	Percentage	Bootstrap 95% CI	
				LLCI	ULCI
Total effect	SC → MPA	–0.672	100.000%	–0.719	–0.625
Direct effect	SC → MPA	–0.392	58.333%	–0.449	–0.335
Indirect effect	SC → FNE → MPA	–0.280	41.667%	–0.326	–0.233

*N* = 971. SC, Self-compassion; MPA, Music Performance Anxiety; FNE, Fear of Negative Evaluation.

### 4.5 Moderation effect analysis

**FIGURE 2:**
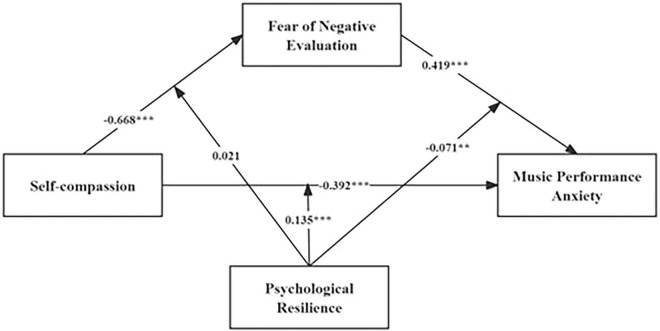
The mediated moderation model tested in the present study. **p < 0.05, ***p < 0.001.

Using Model 59 of the PROCESS macro (version 4.2) in SPSS, we tested the moderated mediation model. The results are summarized in Figure 2 and Table 7.

**TABLE 7:** The moderation effect of psychological resilience.

Outcome variable	Predictor variable	β	SE	*T*	Bootstrap 95% CI	
					LLCI	ULCI
FNE	SC × PR	0.021	0.021	0.100	–0.039	0.043
MPA	SC × PR	0.135	0.026	5.176***	0.084	0.186
	FNE × PR	–0.071	0.028	–2.594**	–0.125	–0.017

*N* = 971.

**p < 0.01;

***p < 0.001. FNE, Fear of Negative Evaluation; SC, Self-compassion; PR, Psychological Resilience; MPA, Music Performance Anxiety.

As shown in Figure 2 and Table 7, we further examined the moderating role of PR in the model. The results indicated that PR significantly moderated the association between SC and MPA (β = 0.135, *p* < 0.001) and also significantly moderated the association between FNE and MPA (β = –0.071, *p* = 0.009). However, PR did not significantly moderate the association between SC and FNE (β = 0.021, *p* = 0.920).

**FIGURE 3:**
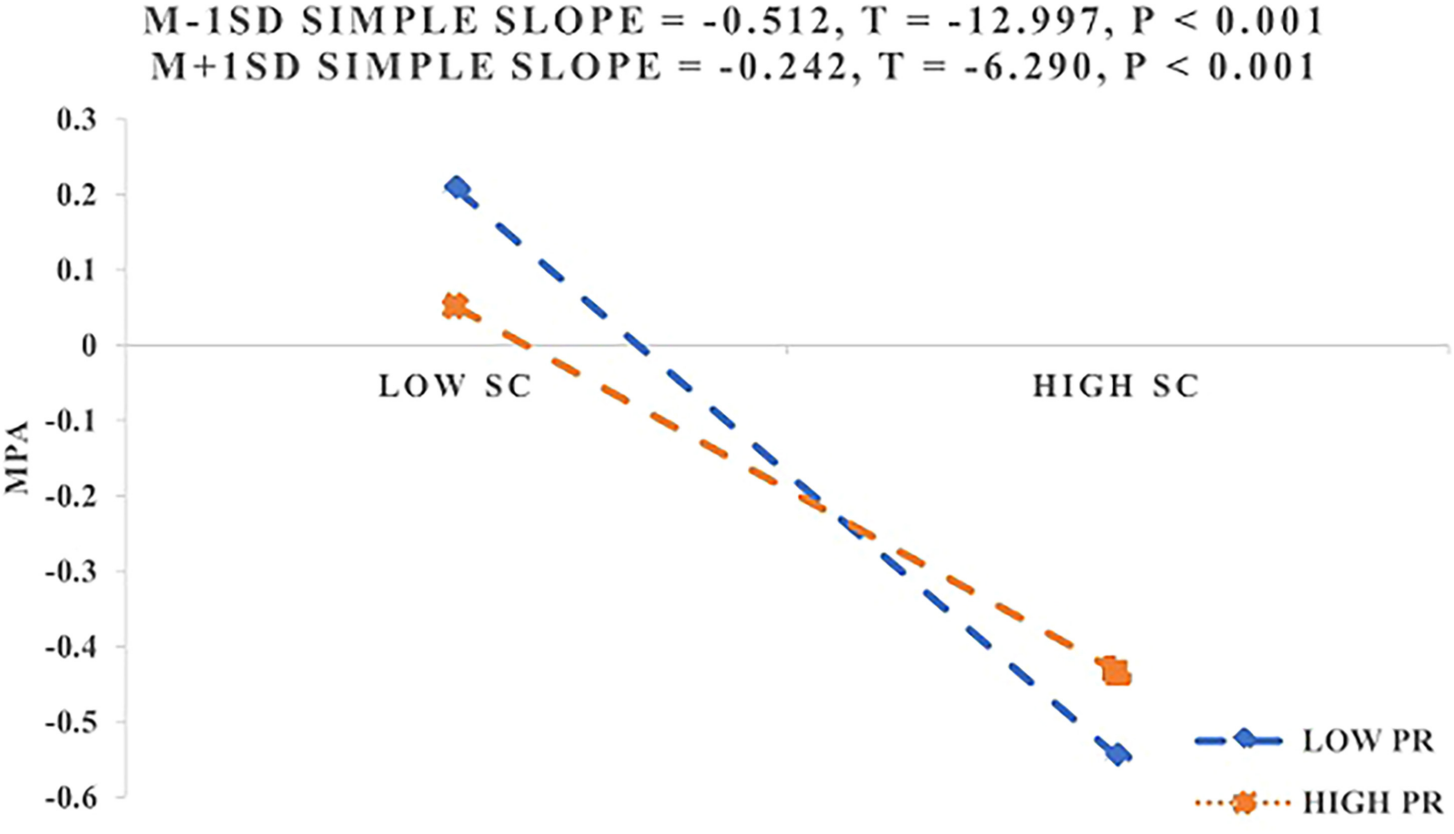
Moderating role of PR between SC and MPA.

We conducted a Johnson–Neyman analysis to better understand the moderating effect of PR on the relationship between SC and MPA. The results revealed that SC had a significant effect when PR was lower than 1.954 SD, encompassing 98.76% of the observed resilience scores. When PR exceeded this cutoff, the effect became non-significant. In addition, simple slope analyses across different levels of PR (Figure 3). PR moderated the relationship between SC and MPA, such that the negative association between SC and MPA was stronger at lower levels of PR and weakened as PR increased. These results do not provide support for H3a.

**FIGURE 4:**
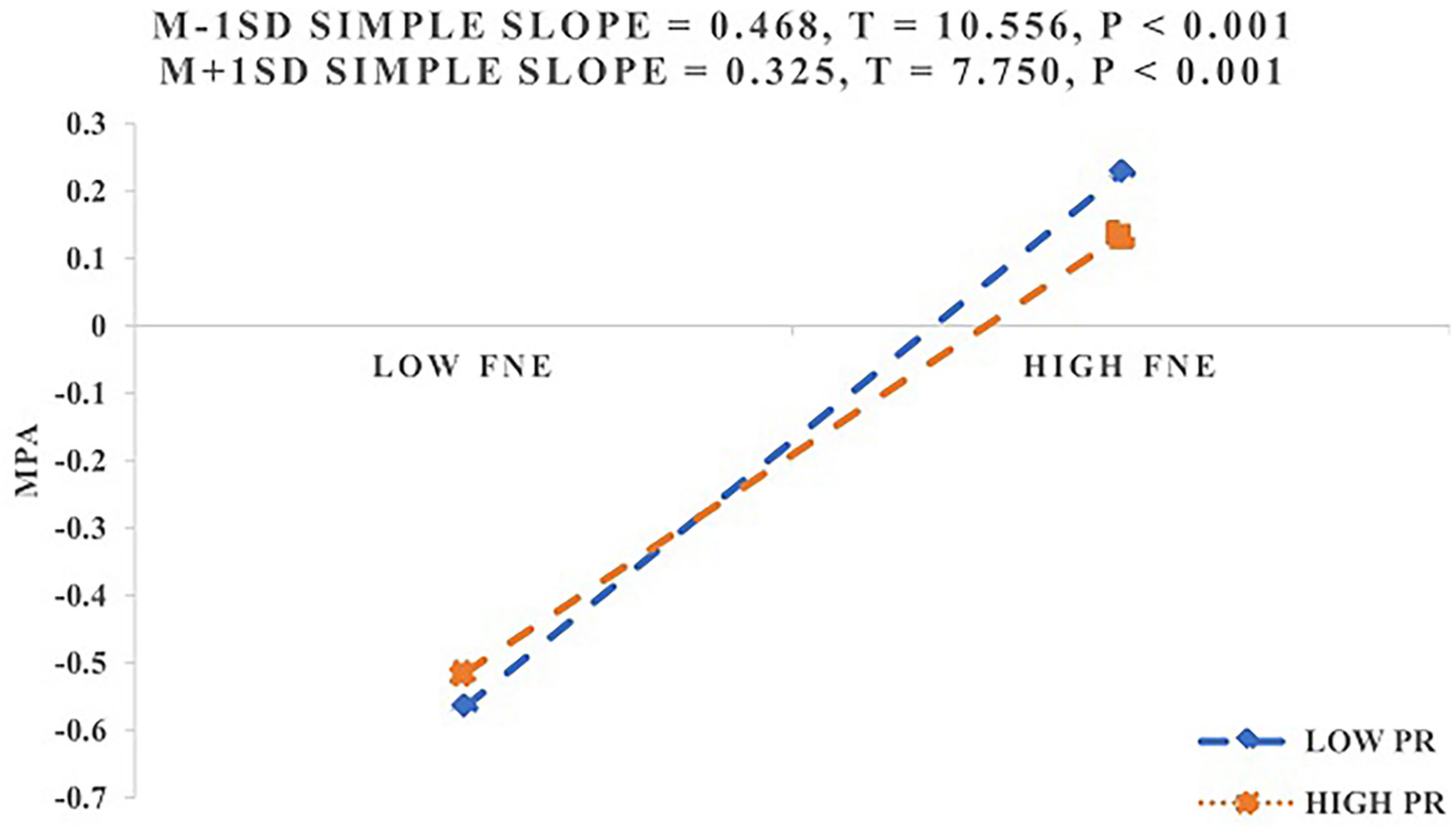
Moderating role of PR between FNE and MPA.

The Johnson–Neyman analysis revealed that PR significantly moderated the relationship between FNE and MPA throughout the entire observed range of FNE. The effect remained significant for all values of PR, and no transition point emerged within the observed data. Additionally, simple slope analyses (Figure 4) indicated that among students with lower PR, FNE was strongly and positively associated with MPA (β *_*simple*_* = 0.468, *p* < 0.001). Among students with higher PR, although the association remained significant, it was comparatively weaker (β *_*simple*_* = 0.325, *p* < 0.001). These findings support H3c.

## 5 Discussion

To examine the association between SC and MPA, this study drew on emotion regulation theory (Gross, 2002) and the risk–protection framework (Fergus and Zimmerman, 2005) and tested the proposed hypotheses using cross-sectional data from 971 college students majoring in music performance. The findings are discussed below.

First, the study showed a significant negative association between SC and MPA, indicating that higher levels of SC were associated with lower levels of anxiety during performance. This finding aligns with the work of Farley and Kelley (2023). Emotion regulation theory suggests that individuals can alleviate emotional distress and enhance psychological adjustment by using different emotion regulation strategies when facing negative emotions (Gross, 2002). SC may be understood as relating to a non-judgmental and accepting stance toward one’s difficulties and shortcomings (Diedrich et al., 2014). In the demanding and high-pressure context of music performance, students who respond to mistakes and challenges with SC are less likely to experience excessive emotional depletion and the buildup of anxiety (Han and Kim, 2023). Rather than engaging in harsh self-criticism or escalating worry, SC helps students recognize that mistakes are common and do not define personal failure; instead, they represent opportunities for growth.

Second, the study showed that FNE mediated in the association between SC and MPA, with the mediating effect accounting for 41.667% of the total effect. This indicates that SC was associated with lower MPA partly through reduced FNE, consistent with Huang and Wang (2024) and Kenny et al. (2013). SC may help individuals become less preoccupied with the possibility of failure or negative judgments (Neff, 2003). In music performance settings, performers often worry about how others will evaluate their abilities, which may contribute to heightened anxiety. Students with higher SC tend to approach their mistakes more forgivingly and avoid interpreting them as personal shortcomings (Werner et al., 2012). Consequently, SC is associated with lower sensitivity to negative evaluation. MPA frequently arises from intense concerns about others’ judgments. During performances, students may focus excessively on their own behavior and feel pressure regarding their perceived competence and technical skill. Research indicates that FNE is strongly associated with anxiety in performance situations; those with higher FNE tend to experience more anxiety (Gómez-López and Sánchez-Cabrero, 2023). Thus, FNE may serve as an important explanatory pathway in the association between concerns about evaluation and tension and unease during performance. Accordingly, lower FNE may partly account for the association between SC and lower performance anxiety. Among individuals with higher SC, acceptance of imperfection and mistakes is more common, which may correspond to a reduced fear of others’ evaluations and, ultimately, lower levels of anxiety.

Finally, the study showed that PR moderated the associations between SC and MPA, and between FNE and MPA. However, PR did not moderate the association between SC and FNE. First, PR significantly moderated the association between SC and MPA, indicating that the negative association between SC and anxiety became weaker as resilience increased. Although this pattern does not fully align with common expectations, one possible explanation is that PR may function as a boundary condition in the association between SC and MPA rather than as a uniformly additive protective resource. When individuals already possess strong emotional regulation resources, the additional benefit of SC may be reduced (Troy et al., 2023). Consistent with the risk–protection framework, protective factors do not always exert linear additive effects; instead, some may complement or even substitute one another (Fergus and Zimmerman, 2005). Thus, for individuals with high PR, the weaker association between SC and anxiety may reflect differences in the use of self-regulatory resources rather than a resource saturation effect. More resilient individuals may rely more on other resilience-related resources that are beneficial in coping with MPA, and SC may therefore have a less prominent role in their self-regulation of anxiety.

Second, PR moderated the association between FNE and MPA, showing a buffering pattern. This suggests that the positive association between FNE and MPA was weaker at higher levels of PR. The observed pattern is consistent with earlier research findings and with the risk–protection framework (Fergus and Zimmerman, 2005). Students with greater PR are more inclined to use positive reappraisal, acceptance, and flexible coping strategies when encountering performance-related stressors, which may reduce the emotional impact of evaluative threats (Tugade and Fredrickson, 2004). Furthermore, those with higher PR tend to sustain emotional balance and show reduced anxiety when facing social threats and judgment pressures (Hu et al., 2015). This finding is consistent with the risk–protection model (Fergus and Zimmerman, 2005), highlighting PR as a crucial protective factor that may weaken the strength of the association between FNE and MPA in the face of external stressors, such as fear of social evaluation. However, PR did not moderate the association between SC and FNE. This finding may suggest that the relationship between SC and FNE is relatively direct. The acceptance and understanding embedded in SC may already be sufficient to be associated with lower sensitivity to negative evaluation, leaving limited room for additional buffering from resilience. Another possible interpretation is that FNE represents a relatively intense emotional–cognitive reaction that may require stronger or more specific regulatory resources beyond PR. Under such circumstances, resilience alone may be insufficient to significantly alter the association.

## 6 Implications and limitations

### 6.1 Theoretical implications

First, this study extends the theoretical understanding of SC by showing that it is associated not only with emotion regulation but also with lower MPA through lower levels of FNE. This finding suggests that SC may help individuals lessen excessive concerns about failure in high-pressure contexts and adopt a more accepting self-attitude, which corresponds to reduced fear of others’ evaluations. Taken together, these findings support the application of emotion regulation theory (Gross, 2002) to music performance settings by suggesting that, when performers face audience scrutiny, fear of mistakes, and evaluative pressure, SC may be associated with lower FNE and, in turn, lower MPA. In this way, emotion regulation theory (Gross, 2002) provides a useful framework for understanding how self-directed acceptance may relate to anxiety experiences in performance-based artistic contexts.

Second, the study highlights the mediating role of FNE in the association between SC and MPA. We found that SC relates to lower levels of MPA indirectly through its association with reduced FNE, providing a new perspective on the underlying psychological processes linked to anxiety. As an indicator of individuals’ sensitivity to external judgment, FNE may help explain why some performers experience heightened anxiety despite possessing other emotion regulation strategies. This result provides further insight into how various psychological factors interact in the context of emotion regulation.

Finally, by incorporating PR into the risk–protection framework (Fergus and Zimmerman, 2005), this study examined its role as an important internal protective resource in the regulation of anxiety. The results showed that PR moderated the associations between SC and MPA and between FNE and MPA, but showed no moderating effect on the connection between SC and FNE. This pattern suggests that PR appears as a protective resource of the MPA, which also moderates the previous relationships with the expected effect, but that, however, PR does not influence how the SC is related to the fear of negative evaluation. This highlights the importance of considering PR as a boundary condition in pathways related to MPA, particularly among individuals in artistic performance contexts, and shows how resilience may shape the strength of specific associations with MPA under high-pressure conditions. These findings provide further empirical support for PR as a moderating emotional resource and offer a useful basis for future intervention research.

### 6.2 Practical implications

First, this research underscores the importance of SC in easing MPA, mainly by decreasing FNE. This finding offers valuable insights for music educators and mental health practitioners. It suggests that cultivating SC in music performance training may enable students to cope with performance-related stress more effectively and experience lower levels of anxiety. Intervention programs for music majors may incorporate SC–based training to support students’ emotional resilience, strengthen their confidence, and ultimately help them better demonstrate the quality of their work during performance, while also supporting their psychological wellbeing.

Second, the study found that PR functioned as a protective factor that moderated the association between SC and MPA. This result provides theoretical guidance for educators and counselors designing psychological support programs. Fostering PR may strengthen students’ adaptive capacities when they encounter performance-related stressors. Additionally, because PR is linked to lower FNE, combining resilience-building strategies with emotion regulation techniques—such as SC training—may help students improve their emotional management skills and mitigate the negative influence of anxiety.

Finally, students majoring in music performance often encounter considerable academic and performance-related pressure, which may contribute to anxiety, depressive symptoms, and other mental health challenges. These difficulties may hinder their academic progress and long-term career development. This study offers practical implications for mental health interventions in artistic education, particularly in reducing performance anxiety and FNE. By integrating emotion regulation strategies such as SC and PR into arts training programs, educators can provide more comprehensive psychological support. Such efforts may help students maintain a healthier mental state in high-pressure environments and enhance their academic performance and professional confidence.

### 6.3 Limitations and future research directions

First, this study adopted a cross-sectional design. Although the findings revealed associations among SC, FNE, PR, and MPA, the design does not allow for conclusions about causality. Cross-sectional data can demonstrate correlations but cannot clarify the directionality of the underlying causal processes. Future studies could explore longitudinal designs to investigate emotional changes over time, providing a clearer understanding of how SC, PR, and performance anxiety are related and how they may affect changes in anxiety in the long run.

Second, the study used self-report questionnaires, where participants evaluated their own emotions and behaviors when reporting anxiety, FNE, and related variables. Self-report methods may be affected by social desirability or recall bias, as individuals might provide responses that reflect social expectations or idealized versions of their emotional experiences. Moreover, emotional states and behavioral responses may fluctuate across different contexts. To improve the reliability and accuracy of findings, future studies could employ a variety of data collection methods, including behavioral, experimental paradigms, and physiological measures.

In addition, the sample in this study was predominantly female, with relatively few male participants. Although this gender distribution reflects the actual demographic pattern of music performance majors in China, but the imbalance could restrict the broader applicability of the findings. To examine whether gender moderates the relationships between SC, FNE, and MPA, future studies should ensure a balanced gender composition. Considering potential gender differences in emotion regulation strategies and anxiety expression, subgroup analyses may provide further insight into whether SC operates differently for male and female students.

Finally, the participants were Chinese college students majoring in music performance. Cultural factors may shape the expression of SC, FNE, and anxiety. Future studies could include a more culturally diverse sample to examine how MPA is managed across different cultural contexts. Such comparisons may be especially meaningful when examining collectivistic versus individualistic cultures, as the use and effectiveness of emotion regulation strategies may differ substantially across cultural contexts.

## 7 Conclusion

Drawing on emotion regulation theory (Gross, 2002) and the risk–protection framework (Fergus and Zimmerman, 2005), this study examined factors associated with MPA among college students majoring in music performance, as well as the moderating role of PR. Using survey data from 971 students, the study found a significant negative association between SC and MPA. FNE served as a partial mediator of this association, accounting for 41.667% of the total effect. In addition, PR significantly moderated two pathways: the association between SC and MPA, and the association between FNE and MPA. Specifically, higher PR corresponded to a weaker negative association between SC and MPA, and a weaker positive association between FNE and MPA. These findings offer new theoretical insights for understanding and addressing MPA and provide practical implications for supporting the mental health and professional advancement of students in the arts. Despite the valuable insights, this study is limited by its cross-sectional design and reliance on self-report measures. Future studies could extend these findings by adopting longitudinal approaches, integrating multiple assessment techniques, and exploring the mechanisms highlighted in this research.

## Data Availability

The datasets presented in this study can be found in online repositories. The names of the repository/repositories and accession number(s) can be found in the article/supplementary material.

## Footnotes

1
https://www.wjx.cn/
